# Disparities in Health Financing Allocation among Infectious Diseases in Ebola Virus Disease (EVD)-Affected Countries, 2005–2017

**DOI:** 10.3390/healthcare10020179

**Published:** 2022-01-18

**Authors:** Kazuki Shimizu, Francesco Checchi, Abdihamid Warsame

**Affiliations:** 1 Department of Health Policy, London School of Economics and Political Science, Cowdray House, Houghton Street, London WC2A 2AE, UK; 2 Faculty of Public Health and Policy, London School of Hygiene and Tropical Medicine, Keppel Street, London WC1E 7HT, UK; 3 Health in Humanitarian Crises Centre, London School of Hygiene and Tropical Medicine, Keppel Street, London WC1E 7HT, UK; Francesco.Checchi@lshtm.ac.uk (F.C.); Abdihamid.Warsame@lshtm.ac.uk (A.W.); 4 Tokyo Foundation for Policy Research, Roppongi Grand Tower 34F, 3-2-1 Roppongi, Minato-ku, Tokyo 106-6234, Japan; 5 Faculty of Epidemiology and Population Health, London School of Hygiene and Tropical Medicine, Keppel Street, London WC1E 7HT, UK

**Keywords:** development assistance for health, health financing, HIV/AIDS, malaria, tuberculosis, Ebola virus disease, vaccine-preventable diseases, health emergency, West Africa, Democratic Republic of Congo

## Abstract

The Ebola virus disease (EVD) outbreaks impacted the population health due to overstretched health systems and disrupted essential health services. Despite a call to achieve equal financial allocation depending on public health needs, there has been scant examination of the fairness of investment among infectious diseases. This study analyzes the extent to which equitable development assistance for health (DAH) has been provided in accordance with disease burden in EVD-affected countries. Estimates of disability-adjusted life years (DALYs) in the Global Burden of Disease (GBD) Study 2017 and DAH Database 1990–2019 in 2005–2017 were analyzed by disease category: vaccine-preventable diseases (VPDs), HIV/AIDS, malaria, tuberculosis, and EVD. HIV/AIDS generally recorded higher ratios of DAH per DALYs (DAH/DALYs). Malaria and tuberculosis showed different trends by country, and VPDs generally presented lower ratios. In West Africa in 2013–2016, DAH/DALYs surged in EVD and fluctuated in HIV/AIDS and malaria. Tuberculosis and VPDs consistently recorded lower ratios. To achieve the risk reduction during and after health emergencies, optimal funding allocation between diseases based on the disease burden is warranted in the pre-emergency period, along with measurement of immediate health needs of populations in real-time during an emergency.

## 1. Introduction

Since its discovery in 1976, the Ebola virus disease (EVD) has been a serious public health threat. Historically, all EVD outbreaks originated in Africa, and according to the Center for Disease Control (CDC) classification, African countries have experienced 19 EVD outbreaks between 2000 and 2020, of which eight occurred in the Democratic Republic of the Congo (DRC) [1]. The global attention toward EVD has surged over the past 10 years. In particular, the 2013–2016 EVD outbreak in West Africa became the largest EVD outbreak in history, recording 28,652 cases and 11,325 deaths (case fatality risk: 39.5%) [2]. The 2018–2020 EVD outbreak in the DRC and Uganda subsequently became the second largest one in the history, with 3480 cases and 2287 deaths (case fatality risk: 65.7%) [3]. The outbreak was recognized as one of the “most complex health emergencies” in the world [4], as it became the first EVD outbreak occurring in a conflict zone [5]; military violence and political instability have been acknowledged as exacerbating the outbreak [6].

EVD outbreaks significantly impacted population health. In West Africa, the EVD outbreak substantially impacted overall mortality [7]. The healthcare systems, which were fragile before the EVD outbreak, were overwhelmed [8], and there have been access interruptions for the test, diagnosis, and treatment of the “big three” diseases: HIV/AIDS, tuberculosis, and malaria [9,10,11,12,13,14,15,16,17]. Simultaneously, health services for non-communicable diseases (NCDs) and maternal and child health were significantly affected [18,19,20,21], and the outbreak heavily impacted mental health [22,23]. The immunization programs on vaccine-preventable diseases (VPDs) were likewise disrupted, causing the resurgence of VPDs during and after the EVD outbreak [24,25,26,27,28,29]. During the EVD outbreak in 2018–2020, the DRC faced the burden of multiple outbreaks of VPDs, such as measles and cholera [30]. Although a vaccination campaign had been conducted, there have been 12,000 cholera cases since January 2019 [31]. The threat of measles is substantial; in 2019–2020, 375,960 cases and 6941 deaths were reported. Most cases were of children aged 5 years and below, and the death toll exceeded that of EVD [32]. Furthermore, the burden of endemic diseases, such as HIV/AIDS, tuberculosis, and malaria, is still immense. Although some programs, including the free care policy, might have slightly contributed to mitigating EVD’s impact [33], to what extent the EVD outbreak has affected population health remains the subject of ongoing research.

To accelerate responses to outbreaks and maintain essential health services, sufficient funding for healthcare is of the utmost importance. It has been suggested that achieving global equal financial allocation depending on public health needs (i.e., recipients’ disease burden) is critical, and this campaign includes development assistance for health (DAH) [34,35,36,37,38,39,40]. Prior to stagnant DAH growth from 2013 to present, there was an overall increasing trend of DAH between 1990 and 2013. However, this rise largely stemmed from the increase of DAH associated with the United Nation’s Millennium Development Goals (MDGs) rather than in response to the precise analysis of disease burden [41]. For example, prevention of and treatment methods for neglected tropical diseases (NTDs), one of the most significant burdens to public health, have received relatively low investment [39]. Although demographic changes suggest that disease burden among elderly people will increase, a large proportion of current DAH targets individuals aged below 60 years [38]. The disproportionate distribution of DAH for NCDs, particularly mental health, has begun to receive significant attention [42]. Lastly, a disparity in funding between fragile and non-fragile states [43,44], or the regional variation of DAH [37], has been documented.

Whereas significant research has compared DAH and disease burden [34,35,36,37,38,39,40], there has been scant examination of the fairness of investment among infectious diseases and of the discrepancy between health financing and disease burden in health emergencies in particular. Here, we retrospectively analyze transitional changes of DAH and disease burden resulting from VPDs, HIV/AIDS, malaria, tuberculosis, and EVD to inform public health policy regarding the extent to which equitable DAH has been provided in accordance with disease burden in EVD-affected countries, clarify how DAH has been aligned with disease burden, and contribute evidence for filling gaps of knowledge on disparities of financial allocation among infectious disease outbreaks.

## 2. Materials and Methods

### 2.1. Selected Countries and Study Period

In this study, we selected four countries as Ebola-affected countries: Guinea, Liberia, Sierra Leone, and DRC, as these heavily necessitated humanitarian assistance during the EVD outbreaks. Due to the fact that 15 most recent EVD outbreaks have occurred after the year 2005, the study period has been set between 2005 and 2017 to give the contemporary and latest assessment of disease-specific funding from available datasets.

### 2.2. Data

To satisfy the objectives of this study, VPDs, HIV/AIDS, malaria, tuberculosis, and EVDs have been compared. VPDs are comprised of twelve diseases: pneumococcal meningitis; H influenzae type B (Hib) meningitis; diphtheria; whooping cough; tetanus; measles; acute hepatitis B; rotavirus; pneumococcal pneumonia; H influenzae type B (Hib) pneumonia; poliomyelitis; and rubella. This selection is based on the definition of Financing Global Health 2018 datasets, and the search strategy used for calculating DAH for child and newborn vaccines were referenced from the User Guide [45,46].

DAH is composed of financial and in-kind resources that originate in international development agencies and are disbursed to low- and middle-income countries (LMICs) for maintaining and improving population health [47]. To analyze the evolution of DAH in EVD-affected countries, the data were extracted from the Development Assistance for Health Database 1990–2019 [45]. DAH specific to each disease was denoted as below by following the definition of the Institute for Health Metrics and Evaluation (IHME) DAH Database (2019) User Guide [45], and the amount of DAH extracted between 2005 and 2017 was categorized by disease. The supplementary methods annex of Financing Global Health 2018 demonstrated that dollars were deflated by the US GDP deflator, which is specific to the reporting year, and keyword searches were conducted for concluding “a subset of global health channels that provide project-level data with project titles or descriptions [46]”. Data sources of DAH are summarized in Table 1.

To measure the burden of diseases, injuries, and risk factors on population health, the concept of disability-adjusted life years (DALYs) has been introduced [48,49], and it is widely used by international organizations, including WHO, for quantifying disease burden [50]. DALYs are useful in that both traditional epidemiological data on mortality and information about the loss of quality of life with several value choices are combined, and the disease burden is quantified by taking time as a unit [48,49]. Currently, results of the Global Burden of Disease (GBD) Study have been widely used at national, regional, and global levels for strategically considering the resource allocation in various public health programs [51]. To analyze the evolution of DALYs in EVD-affected countries, both the all-cause DALYs and the DALYs specific to HIV/AIDS, malaria, tuberculosis, and EVD between 2005 and 2017 were extracted from the Global Burden of Disease Collaborative Network and arranged by following the GBD 2017 Online Tools Overview [52]. The age group was set as all ages (age group ID: 22), and the cause ID is presented in Table 2.

Regarding VPDs, DALYs specific to diseases that are classified into child and newborn vaccines in DAH were extracted (Table 3).

To follow the definition of a child by IHME [47], DALYs were extracted by specifying the age group under five years (age group ID: 1) and calculated by the following formula:

(1) $${\mathrm{DALYs}}_{\left(\mathrm{VPDs}\right)}=\sum_{m}{\mathrm{DALYs}}_{\left(\mathrm{Cause}\;m\right)}+\sum_{n}{\mathrm{DALYs}}_{\left(\mathrm{Etiology}\;n\right)}$$

### 2.3. Data Analysis

The absolute DAH and DALYs over time were calculated by year and shown by disease in EVD-affected countries. Data in Guinea, Liberia, and Sierra Leone were also compiled as West Africa region. To conduct these analyses, only the point estimated data were used. To match DAH with DALYs, the ratio of DAH to DALYs (unit: USD/DALYs) is represented as:

(2) $$\frac{\sum_{\mathrm{ij}}{\mathrm{DAH}}_{\mathrm{ij}}}{\sum_{\mathrm{ij}}{\mathrm{DALYs}}_{\mathrm{ij}}}$$

where i is the country and j is the type of disease. Namely, ratios were calculated by total DAH and all-cause DALYs, as well as those specific to VPDs, HIV/AIDS, malaria, and tuberculosis, and EVD, and aggregated by region and country. The point estimates and 95% uncertainty intervals of DALYs data were used for the analysis.

## 3. Results

### 3.1. DAH for EVD-Affected Countries

**Figure 1:**
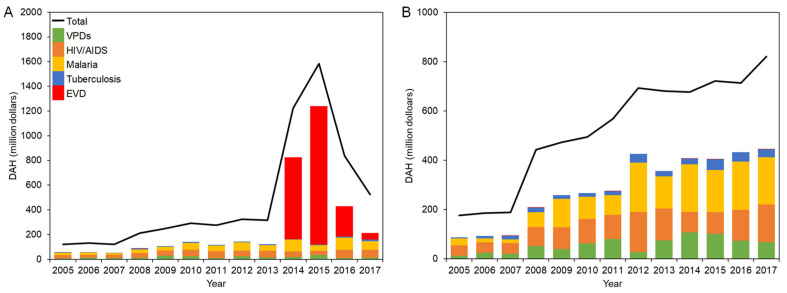
DAH for EVD-affected countries in 2005–2017: (**A**) West Africa; (**B**) DRC.

DAH for countries in West Africa and DRC is shown in Figure 1A,B, respectively.

In 2005, the total DAH for West Africa was USD 121 million, and the two dominant categories, HIV/AIDS (22.8%) and malaria (16.5%), were followed by VPDs (5.6%). While minor decreases were observed in 2011 and 2013, there was an upward tendency of total DAH on the whole, which exceeded USD 300 million in 2012, and HIV/AIDS and malaria continuously comprised the larger proportion. It quickly soared to USD 1.22 billion in 2014 and finally peaked at USD 1.58 billion in 2015 before plummeting to USD 525 million in 2017, due to a surge of DAH for EVD from USD 662 million in 2014 to USD 1.12 billion in 2015, which constituted 70.7% of total DAH for that year.

The breakdown of DAH for each country in West Africa and exact values are presented in Supplementary Figure S1 and Supplementary Table S1, respectively. In 2005, the total DAH for Liberia was only USD 25.0 million, which was below compared to DAH received by Guinea (USD 44.8 million) and less than half of Sierra Leone (USD 51.1 million). However, it has jumped up to USD 82.4 million in 2008, which was higher compared to the other two countries, and the trend continued by 2013. Among the disease category, HIV/AIDS was more funded compared in the pre-emergency period in Sierra Leone except in 2006, while in Liberia, the amount of DAH for malaria generally exceeded DAH for HIV/AIDS, except for some changes in 2007 and 2013. DRC received higher investment through DAH compared to countries in West Africa. The total DAH for the DRC continued to grow to USD 821 million in 2017. In 2005–2008, investment for HIV/AIDS accounted for more than 20% of total DAH. DAH for malaria dramatically increased to USD 199 million in 2012, which constituted 28.7% of total DAH, and continuously exceeded that DAH for HIV/AIDS in 2012–2017. DAH for VPDs increased to USD 108 million in 2014. However, a marked decline was observed afterward and fell to USD 68.7 million in 2017. DAH for tuberculosis was limited to less than USD 4.20 million in 2005, and as time went by, it amounted to USD 43.8 million (6.1%) at its maximum in 2015. Tuberculosis consistently occupied less than 8% of total DAH between 2005 and 2017. The DRC experienced several EVD outbreaks during the study period, and DAH for EVD was recorded for responding to outbreaks. However, EVD does not have an overwhelming impact on total DAH.

### 3.2. DALYs in EVD-Affected Countries

**Figure 2:**
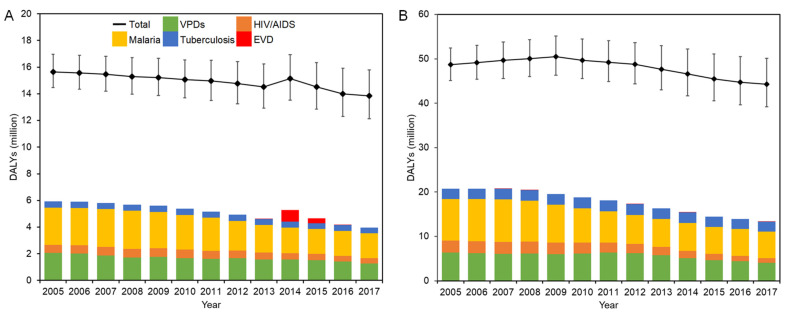
DALYs in EVD-affected countries in 2005–2017: (**A**) West Africa; (**B**) DRC. Solid squares represent the point estimates of all-cause DALYs, and whiskers extend to the upper and lower 95% confidence intervals.

The evolution of DALYs in West Africa and DRC is shown in Figure 2A,B, respectively.

There was a steady drop in all-cause DALYs in West Africa except in 2014, reaching 13.9 million in 2017. Malaria accounted for the largest proportion of DALYs with as many as 2.85 million DALYs, peaking at 18.7% of all-cause DALYs recorded in 2008. DALYs in VPDs were over 2.07 million (13.3%) in 2005, and there has been a steady decrease over the years, declining to 1.28 million (9.2%) in 2017. DALYs of tuberculosis steadily decreased from 2005, finally reaching 0.43 million in 2017. During the EVD outbreak between 2013 and 2016, while the epidemiological burden of EVD significantly increased, there was a steady fall in DALYs of other categories.

The breakdown of DALYs in each country in West Africa and exact values are presented in Supplementary Figure S2 and Supplementary Table S2, respectively. Guinea consistently recorded the highest total DALYs among the three countries, which was followed by Sierra Leone. The lowest total disease burden was recorded in Liberia. In Sierra Leone, malaria consistently recorded the highest disease burden among the selected diseases, which was followed by VPDs. A similar trend was observed in Guinea in 2005–2014 and 2017. On the contrary, the disease burden of VPDs has started to exceed that of malaria since 2013. During the emergency period, each country showed different trends. While the disease burden by EVD was recorded the highest in Liberia in 2014, it was the lowest amount in the selected disease category in Guinea in 2014–2015.

In DRC, the all-cause DALYs decreased by more than 4 million during the study period. Among the five disease categories, malaria constituted the largest proportion accounting for 19.3% of all-cause DALYs in 2005. Although this decreased to 6.00 million in 2017, it still dominated 13.5% of total DALYs. VPDs recorded the second-highest disease burden, and DALYs in VPDs stayed at a level of 6.0–6.4 million between 2005 and 2012. This was accompanied by a steady decrease over the years after 2012, finally declining to 4.07 million (9.2%) in 2017. DALYs in HIV/AIDS made up 5.5% (2.66 million) of all-cause DALYs in 2005. While this figure hovered around 2.6 million in 2005–2008, it steadily decreased to 1.00 million (2.3%) by the end of 2017. There was relatively little change in DALYs in tuberculosis, which remained at around 2.2–2.5 million between 2005 and 2017, accounting for roughly 5% of all-cause DALYs during the study period.

### 3.3. DAH per DALYs by Disease

**Figure 3:**
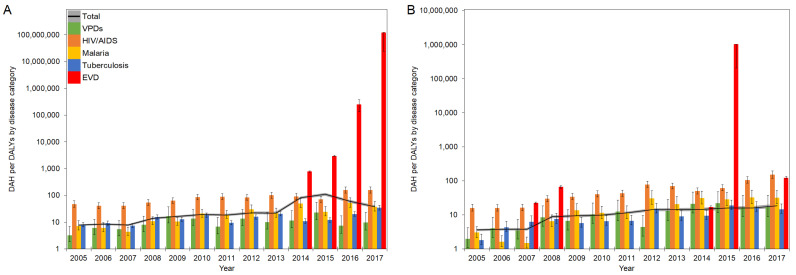
DAH/DALYs in EVD-affected countries in 2005–2017: (**A**) West Africa; (**B**) DRC. The black line represents the total DAH/DALYs, and the gray area is extended to the upper and lower 95% confidence intervals based on variations of DALYs. Colored bars represent the DAH/DALYs by disease category. Whiskers extend to the upper and lower 95% confidence intervals based on variations of DALYs. A common logarithmic scale is used on the vertical axis.

An evolution of DAH per DALYs (DAH/DALYs) in West Africa and DRC in 2005–2017 is presented in Figure 3A,B, respectively.

Overall, the total DAH per all-cause DALYs (total DAH/DALYs) in West Africa showed a moderate but steady increase from 2005 to 2012. After a small decrease in 2013, there was a steep increase to 109.6 DAH/DALY in 2015 before a sharp decline from 2016 onward. DAH/DALYs of HIV/AIDS gradually increased over time, followed by a fall in 2015 and rebound in 2017, and they recorded higher ratios compared to total DAH/DALYs except in 2015, and. In contrast, DAH/DALYs of VPDs, malaria, and tuberculosis were generally lower than total DAH/DALYs over the years, except for malaria in 2012. However, the DAH/DALYs of malaria showed relatively higher ratios than those of VPDs and tuberculosis. During the EVD outbreak between 2013–2016, the DAH/DALYs in HIV/AIDS, malaria, and tuberculosis experienced declines, while the DAH/DALYs in VPDs showed rapid increases in 2015, followed by a return to their original levels. In DRC, there was a gradual increase of total DAH/DALYs over the period, from 3.6 DAH/DALY in 2005 to 18.5 DAH/DALY in 2017. Although DRC experienced multiple EVD outbreaks in 2007, 2008, 2012, 2014, and 2017 and DAH/DALYs specific to EVD significantly increased, especially in 2015 at over 1 million DAH/DALY, they did not significantly influence the trend of DAH/DALYs in total and other infectious diseases.

**Figure 4:**
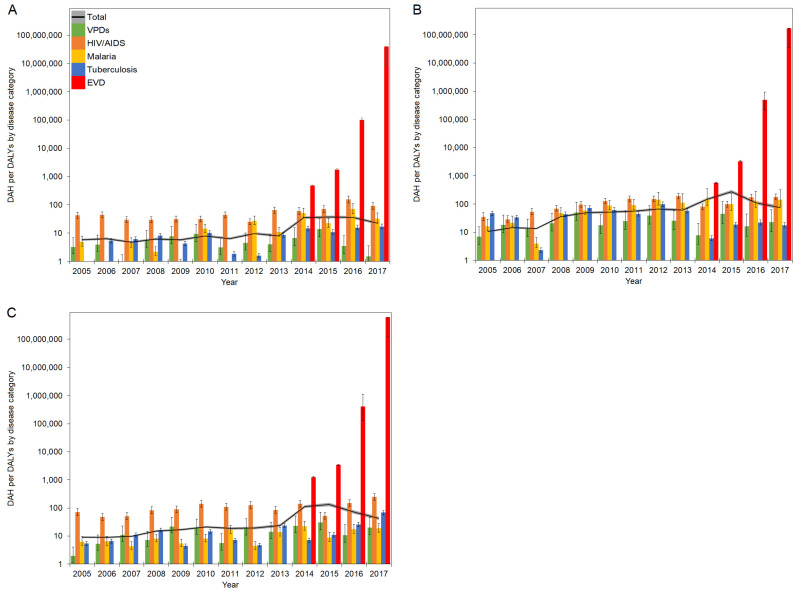
DAH/DALYs in countries in West Africa in 2005–2017: (**A**) Guinea; (**B**) Liberia; (**C**) Sierra Leone. The black line represents the total DAH/DALYs, and the gray area is extended to the upper and lower 95% confidence intervals based on variations of DALYs. Colored bars represent the DAH/DALYs by disease category. Whiskers extend to the upper and lower 95% confidence intervals based on variations of DALYs. A common logarithmic scale is used on the vertical axis.

Furthermore, ratios in West Africa were subdivided into each country. Ratios of DAH per DALYs by diseases in Guinea, Liberia, and Sierra Leone are presented in Figure 4A–C, respectively. Exact values for Figure 3 and Figure 4 are checked in Supplementary Table S3.

The total DAH/DALYs in 2005 ranged from 5.8 in Guinea to 10.5 in Liberia. While the total DAH/DALYs remained below 10 DAH/DALY in Guinea, they generally showed increasing trends in Liberia and Sierra Leone to 2013. During the EVD outbreak, DAH/DALYs in EVD surged in all countries and recorded the highest ratios in 2017, with 39.7 million DAH/DALY in Guinea, 172.5 million DAH/DALY in Liberia, and 603 million DAH/DALY in Sierra Leone. During the EVD outbreak between 2013 and 2016, DAH/DALYs in HIV/AIDS, malaria, tuberculosis, and VPDs generally fluctuated in Liberia and Sierra Leone; on the contrary, DAH/DALYs to HIV/AIDS and tuberculosis remained steady in Guinea. Although VPDs generally recorded the lowest ratios among disease categories in Liberia, DAH/DALYs to VPDs outnumbered those of tuberculosis in 2015 and 2017.

## 4. Discussion

This study presented four major findings. First, while there were slight fluctuations, the total DAH for EVD-affected countries demonstrated an increasing trend both in West Africa and DRC and peaked in 2015 in West Africa. During the EVD outbreak in West Africa, EVD became the most funded disease in 2014–2015, and DAH for other infectious diseases appears to have been disrupted. Second, the all-cause DALYs gradually decreased over the study period, and malaria and VPDs respectively constituted the largest and the second-largest proportions. In both regions, DALYs by disease consistently showed a decreasing trend over the years. In 2014 in West Africa, DALYs of EVD exceeded DALYs of HIV/AIDS and tuberculosis but were less than DALYs of malaria and VPDs. Third, there have been skewed funding allocations in DAH between infectious diseases in the pre-emergency period, compared to their disease burden. DAH/DALYs for HIV/AIDS were generally higher than total DAH/DALYs. However, malaria and tuberculosis showed different trends by country. Over the study period, DAH/DALYs for VPDs were generally lower than total DAH/DALYs in EVD-affected countries. Fourth, DAH was prioritized for EVDs during health emergencies, and DAH for other infectious diseases showed different trends. In 2013–2016 in West Africa, a fluctuation of DAH/DALYs was detected for HIV/AIDS in Liberia and Sierra Leone and for malaria in Guinea and Liberia. However, VPDs and tuberculosis consistently recorded lower ratios than total DAH/DALYs.

### 4.1. Skewed Funding Allocation in Pre-Emergency Period

Our study suggested that tuberculosis and VPDs have recorded relatively lower ratios of DAH/DALYs compared to HIV/AIDS and malaria in the pre-emergency period. The emergence of global targets that specify clear goals and targets regarding HIV/AIDS and malaria might have affected the consideration process of allocating DAH. Especially in the United Nations (UN) Millennium Development Goals (MDGs), tuberculosis was only included among “other diseases” in Target 6C, thus placing disproportionate emphasis on HIV/AIDS and malaria in the health-related development agenda. Although the importance of intervention for VPDs was noted, there has been scant discussion regarding specific diseases that require primary focus. Moreover, in the early 2000s, HIV/AIDS was sublimated as a political and developmental agenda in global public policy, as represented by the 2001 UN General Assembly Special Session on HIV/AIDS and the UN General Assembly Political Declaration on HIV/AIDS in both 2006 and 2011 [53], which could potentially bring the disproportionate DAH allocation between infectious diseases.

In addition, the characteristics of each disease might have contributed to skewing attention toward each infectious disease. Tuberculosis was relatively neglected compared to HIV/AIDS and malaria, as challenges in tuberculosis were featured mainly among HIV co-infected cases. This was different from malaria, which records increasing morbidity and mortality among patients with drug-resistant strains, which are recognized as a substantial threat for controlling its spread. Furthermore, there are differences in these diseases’ clinical characteristics. While malaria could be recognized as an acute illness in a period of days and weeks after infection, tuberculosis typically develops slowly, and symptoms may not emerge until months or years after infection. Even patients infected with latent tuberculosis will not exhibit any symptoms, making it difficult to securitize tuberculosis. On the other hand, VPDs have been continuously conceptualized not as a security agenda but merely as issues in public health, possibly as they were not health threats in many developed countries where vaccination programs are available, skewing the attention as a global political agenda.

Further, differences between EVD-affected countries need to be further scrutinized. In the pre-emergency period in West Africa, each country has shown different trends of DAH/DALYs by disease category. Especially, while the DAH/DALYs of malaria in Liberia consistently exceeded the total DAH/DALYs except for a dip in 2007, the opposite trend was monitored in Sierra Leone. With regard to DAH/DALYs in tuberculosis, Liberia recorded a higher ratio, and the frequency of exceeding the total DAH/DALYs was higher compared to Guinea and Sierra Leone. These findings indicate further investigation of the relatively higher DAH towards malaria and tuberculosis in Liberia.

### 4.2. Funding Prioritization during Health Emergencies

Our study also clarified that EVD was disproportionately earmarked for containment during a health emergency. Insufficient preparedness for health emergencies due to a lack of capacity at the national level might have promoted a vertical approach that invested substantial resources in EVD without adequate analysis of overall public health needs. Moreover, despite public awareness and the serious nature of the threat, there has been a sluggish response to the EVD outbreak, as illustrated by the late declaration of a Public Health Emergency of International Concern [54], which necessitated a vertical approach to visible health threats and dictated unequal resource allocation among diseases, while overlooking the disease burden of endemic diseases and interrupting preventable measures such as vaccination due to health system collapses. DAH/DALYs for diseases that were also threats in EVD-affected countries but have relatively lower risks of transmission for Western countries received far less investment, suggesting the necessity of considering populations in need appropriately.

Regarding other disease categories, while Guinea recorded the higher DAH/DALYs in HIV/AIDS over the total DAH/DALYs even during the EVD outbreaks, similar trends were not observed in Liberia and Sierra Leone, suggesting the necessity of investigating underlying mechanisms between countries. Additionally, although DRC experienced the EVD outbreak in 2014 that recorded 66 cases and 49 deaths, it has been successfully contained within a period of 3 months, which was completely different from the EVD outbreak in West Africa that necessitated year-long responses. The evolution of DAH/DALYs clearly suggested that while the DAH/DALYs in EVD significantly increased for containment in DRC, DAH/DALYs in other categories were not seriously affected due to the quick containment of imminent health emergency.

### 4.3. Utilization of Disease Burden for Health Financing

Although careful examination is required because the overall amount of health spending at the national level is not considered and there are some methodological uncertainties, our study provides a general picture of the limited alignment between DAH for each infectious disease category and each category’s disease burden in EVD-affected countries. Our study calls for the evidence-based management of allocating health financing that is scarce. Although there are always competing priorities, using the data of disease burden is critical for making decisions in prioritization, and it is not only limited to the purpose of DAH. To ensure the openness and transparency in funding priorities, the monitoring system of DAH at the global level, as well as health financing at the national level, must be improved. To build a solid foundation for achieving risk reduction during health emergencies, particularly large outbreaks, combining DAH and disease burden for improving the decision making of prioritization in resource allocation, allocating sufficient and appropriate resourcing based on the health needs of populations in both the pre-emergency period and emergency phase, and scrutinizing responses to health emergencies in real-time and revising them accordingly, is pivotal. To achieve these assignments, strengthening the resilience of health systems and incorporating the provision of essential health services into health emergency response plans is imperative to reduce health risks in the post-emergency period. This study explicitly indicated that some disease categories, especially VPDs, were relatively underfunded, and these must be given more priority, and disparities need to be corrected and managed based on the scientific data.

If the trends observed in this study hold true and considering the impact of the ongoing coronavirus disease 2019 (COVID-19) pandemic, it is likely expected that funding for a COVID-19 response in LMICs will increase significantly, while the prevention of other prevalent diseases that pose higher burdens, especially VPDs, may still lack investment. In fact, the disruption of routine childhood immunization programs due to logistic issues, staff shortages, and reallocation of healthcare resources for COVID-19 may result in an increase in the incidence of and deaths from VPDs [55]. The interruption of supplying antiretroviral therapy drugs and provision of preventive measures will engender additional HIV-related deaths [56]. Recent modeling has also suggested that deaths by malaria and tuberculosis will increase by 36% and 20%, respectively, in areas with higher disease burden if the COVID-19 pandemic disrupts crucial prevention activities and healthcare services [57]. Therefore, actors in global health must make efforts to promptly contain the pandemic and secure sufficient resources for other critical issues in public health. This will help balance the responses toward each disease, thus improving population health. Furthermore, the health emergency must prompt healthcare system reform [58], and both technical challenges that are free from politics and political difficulties require review to maintain essential health services. Comprehensive, multi-sectoral approaches that ensure cost-effectiveness and affordability will promote a positive spillover for addressing the burden of infectious diseases, other disease categories, and health system strengthening.

### 4.4. Limitations

Several limitations should be noted in the present study. First, this analysis used the data of disbursed DAH and did not evaluate how funds are received and spent at a national level. Utilizing the latter data will prove instrumental to assessing action on the ground. Second, this analysis solely relied on the estimated published by IHME. Globally, there are generally several available data sources, including the Creditor Reporting System (CRS), managed by the Organization for Economic Cooperation and Development (OECD) [59], and the Financial Tracking Service (FTS), overseen by the United Nations Office for the Coordination of Humanitarian Affairs (OCHA) [60]. While these sources were not used due to a lack of aggregated data on VPDs and EVD and dispersed data on HIV/AIDS, comparative analysis of DAH allocation based on different data sources may help extract challenges of health financing and ultimately improve the current situation of DAH allocation. Additionally, as this study followed the definition of DAH that is presented by IHME, DAH spent on prevention efforts for each disease category might have been insufficiently addressed. Generally, many DAH-related projects aim at building health systems with a specific focus on health areas. While these investments, which are categorized as “other health systems strengthening” in each category, were analyzed in this study, DAH in the category of HSS/SWAps (health system strengthening and sector-wide approaches) are exempted from the analysis. Third, DALYs are useful tools for measuring population health and have been used as a benchmark for health policy; however, several limitations of DALYs, as summarized by Anand and Hanson [61], and Solberg et al. [62], must always be noted. Simultaneously, continuous evaluation and further scrutinization of the applicability of DALYs for evaluating the equality of DAH allocation will be pivotal. For example, solely relying on the simple ratio of DAH/DALYs could underestimate potential gains attainable from certain financing in a specific period, especially for infectious diseases that are targeted for eradication (e.g., poliomyelitis) and elimination (e.g., neglected tropical diseases, measles). This point could be complemented with other metrics and by broader standpoints. Fourth, scant research has addressed how to use DALYs as an indicator to measure the impact of health emergencies, and this point necessitates further academic discussion. Fifth, due to data availability, DALYs in polio and rubella are not included in this study, which might possibly help overestimate the DAH/DALYs in VPDs. Sixth, this study used the ratio of DAH to DALYs specific to each category. These metrics have already been used in previous research, but an open investigation is necessary to identify better indicators for determining the amount of DAH because estimates of disease burden are crucial but are not the sole input for prioritization. Finally, it is well known that health expenditures at the national level decrease when the allotment of DAH to governments increases [63,64,65], indicating some challenges in monitoring and evaluation [36]. Combining DAH with domestic health expenditures specific to each disease is a potential idea for tackling this limitation, but comprehensive data are required.

Future research must take these limitations into account and address some of the uncertainties of indicators. Although this analysis could not address the evolution of DAH/DALYs in relation to HIV/AIDS, malaria, tuberculosis, and VPDs during the EVD outbreak in the DRC in 2018–2020 because data are still not available to the public, our analysis of DAH/DALY in West Africa may be indicative of funding during health emergencies. Our analysis can support the hypothesis that investment during the EVD outbreak was disproportionately reserved for EVD at the expense of other diseases, particularly VPDs. Measuring those trends during the post-emergency period in West Africa and in the emergency phase in the DRC can be the research topic in the near future. Furthermore, the coronavirus disease 2019 (COVID-19) pandemic will affect population health to a substantial degree, and the amount of DAH is expected to surge over the next several years. Therefore, investigation of DAH/DALYs in COVID-19 and comparison of ratios with other disease categories are potentially valuable research avenues.

## 5. Conclusions

This study has highlighted the evolution of DAH and disease burden in EVD-affected countries both in the pre-emergency period and emergency phase. A skewed funding allocation between infectious diseases in the pre-emergency period was observed. To achieve the risk reduction during and after health emergencies, optimal funding allocation among infectious diseases based on the disease burden is warranted in the pre-emergency period. Additionally, measuring the immediate health needs of populations in real-time will be pivotal. Controlling infectious diseases and the prioritization of funding for each disease is a globally significant agenda and not limited to countries affected by EVD outbreaks. Every country receiving DAH has confronted the same concern and must determine the best strategies for optimal health financing allocation during health emergencies. Our study presents an opportunity to learn from challenges that the EVD outbreak has brought to health financing and prioritization among different infectious diseases. The evidence presented will help all levels of decision-makers to promote evidence-based policymaking for achieving fair allocation of funding among infectious diseases before and during health emergencies.

## Figures and Tables

**Table 1:** DAH data.

Disease Category	Explanation	Variable Name
Total	Total funds for health disbursed from source to channel to recipient country	dah_19
VPDs	Funds for health disbursed from source to channel to recipient country for newborn and child health, disaggregated by vaccines	nch_cnv_dah_19
HIV/AIDS	Funds for health disbursed from source to channel to recipient country for HIV/AIDS	hiv_dah_19
Malaria	Funds for health disbursed from source to channel to recipient country for malaria	mh_dah_19
Tuberculosis	Funds for health disbursed from source to channel to recipient country for tuberculosis	tb_dah_19
EVD	Funds for health disbursed from source to channel to recipient country for other infectious diseases, disaggregated by Ebola	oid_ebz_dah_19

**Table 2:** DALYs data (except VPDs).

Age Group Name	Cause ID	Cause Name	Hierarchy
All ages	294	All causes	Total
	298	HIV/AIDS	A.1.1
	345	Malaria	A.4.1
	297	Tuberculosis	A.2.1
	843	Ebola	A.4.17

**Table 3:** DALYs data (VPDs).

Age Group Name	Cause ID	Cause Name	Hierarchy
Under 5 years	333	Pneumococcal meningitis	A.5.1.1
	334	H influenzae type B meningitis	A.5.1.2
	338	Diphtheria	A.5.3
	339	Whooping cough	A.5.4
	340	Tetanus	A.5.5
	341	Measles	A.5.6
	402	Acute hepatitis B	A.5.8.2
	**Etiology ID**	**Etiology Name**	
	181	Rotavirus	
	188	Pneumococcal pneumonia	
	189	H influenzae type B pneumonia	

As DALYs specific to poliomyelitis and rubella, including congenital rubella syndrome, were not available in the GBD study, their disease burdens were not included in the analysis.

## Data Availability

The data are publicly available at websites cited on references [45,46,52].

## Supplementary Materials

The following supporting information can be downloaded at: https://www.mdpi.com/article/10.3390/healthcare10020179/s1, Figure S1: DAH (thousand dollars) for selected countries in West Africa: (A) Guinea; (B) Liberia; (C) Sierra Leone, Figure S2: DALYs (thousands) in selected countries in West Africa: (A) Guinea; (B) Liberia; (C) Sierra Leone, Table S1: DAH (thousand dollars) for EVD-affected countries, Table S2: DALYs (thousands) in EVD-affected countries, Table S3: DAH/DALYs in EVD-affected countries.
