# Supplementary material for: Disparities in Health Financing Allocation among Infectious Diseases in Ebola Virus Disease (EVD)-Affected Countries, 2005–2017
Source: Healthcare (Basel). 2022 Jan 18;10(2):179. doi: 10.3390/healthcare10020179 (PMC8872520; doi:10.3390/healthcare10020179)
Supplement: Supplementary file 1 [file healthcare-10-00179-s001.zip › shimizu_suptable_9Jan2022.pdf]

Supplementary Table S1: DAH (thousand dollars) for EVD affected countries

A. Guinea

Category	2005	2006	2007	2008	2009	2010	2011	2012	2013	2014	2015	2016	2017
Total	44,759	48,986	36,529	47,091	43,593	59,147	46,691	70,940	57,931	258,105	264,868	251,320	155,340
VPDs	3,635	4,350	875	5,612	7,852	8,823	2,871	4,266	3,587	5,969	12,265	2,928	1,123
HIV/AIDS	12,350	13,525	9,304	9,527	9,958	9,623	12,959	6,966	15,461	13,358	14,760	29,645	16,613
Malaria	5,684	723	5,093	2,546	892	15,873	316	28,143	10,750	47,304	19,185	57,933	26,880
Tuberculosis	20	1,235	1,437	1,895	1,006	2,408	434	368	2,045	3,456	2,556	3,633	3,867
EVD	—	—	—	1	—	—	—	—	—	90,253	162,159	55,434	11,834

B. Liberia

Category	2005	2006	2007	2008	2009	2010	2011	2012	2013	2014	2015	2016	2017
Total	25,044	33,831	31,434	82,417	113,604	120,386	131,451	153,947	139,521	382,699	654,128	241,823	162,785
VPDs	1,779	4,201	2,737	4,028	9,892	3,692	5,233	8,457	5,727	1,739	9,998	3,307	4,370
HIV/AIDS	6,181	5,131	9,444	11,931	15,592	19,330	21,835	19,461	22,801	8,948	10,597	17,665	17,938
Malaria	6,266	8,310	1,479	18,177	20,780	29,151	28,446	34,507	22,381	30,456	19,004	24,172	26,718
Tuberculosis	2,766	1,939	131	2,485	4,145	3,646	2,703	6,075	3,550	367	1,096	1,317	1,037
EVD	—	—	—	2	—	—	—	—	—	211,578	503,454	79,933	14,285

C. Sierra Leone

Category	2005	2006	2007	2008	2009	2010	2011	2012	2013	2014	2015	2016	2017
Total	51,071	49,873	53,805	81,787	91,762	111,867	98,180	100,813	119,760	582,377	665,539	344,283	207,644
VPDs	1,364	3,547	6,496	3,947	11,974	10,547	2,789	9,670	6,591	10,415	12,963	4,182	6,977
HIV/AIDS	9,059	6,965	8,412	14,556	16,313	25,561	19,482	21,590	14,237	22,474	7,809	20,697	29,702
Malaria	7,963	8,232	5,649	10,722	6,851	9,314	18,001	4,347	12,142	18,010	7,225	14,865	16,057
Tuberculosis	951	1,158	1,893	2,808	748	2,468	1,183	773	3,765	1,125	1,688	3,793	9,853
EVD	—	—	—	2	—	—	—	—	—	360,573	454,416	111,246	25,197

D. Democratic Republic of Congo

Category	2005	2006	2007	2008	2009	2010	2011	2012	2013	2014	2015	2016	2017
Total	175,972	185,263	188,969	442,473	472,461	495,120	568,240	692,440	681,314	676,375	721,021	714,014	820,680
VPDs	12,640	25,057	21,133	51,813	40,289	63,518	80,329	27,611	74,507	108,284	102,637	73,250	68,751
HIV/AIDS	42,219	42,461	43,258	77,936	87,802	98,863	98,106	163,371	129,902	81,934	86,946	125,567	151,200
Malaria	28,592	15,639	14,346	60,006	116,288	88,964	79,562	199,013	130,580	193,661	170,539	195,287	192,403
Tuberculosis	4,195	10,173	14,882	18,045	14,032	16,137	16,384	36,477	21,722	22,459	43,799	37,995	32,295
EVD	—	—	464	105	—	—	16	—	—	87	713	—	62

Supplementary Table S2: DALYs (thousands) in EVD affected countries

A. Guinea

Category	2005	2006	2007	2008	2009	2010	2011	2012	2013	2014	2015	2016	2017
Total	7,696	7,673	7,636	7,541	7,504	7,422	7,340	7,275	7,202	7,310	7,129	6,944	6,868
VPDs	1,122	1,105	1,064	970	1,015	934	907	931	883	885	877	827	743
HIV/AIDS	289	304	313	319	318	304	290	276	238	220	210	191	182
Malaria	1,140	1,136	1,147	1,156	1,116	1,118	1,091	1,033	991	921	858	823	823
Tuberculosis	230	233	234	234	233	236	234	232	234	236	234	231	226
EVD	0	0	0	0	0	0	0	0	0	188	91	1	0

B. Liberia

Category	2005	2006	2007	2008	2009	2010	2011	2012	2013	2014	2015	2016	2017
Total	2,377	2,331	2,315	2,304	2,299	2,320	2,363	2,321	2,275	2,614	2,364	2,196	2,162
VPDs	258	231	214	193	196	210	209	219	225	223	222	205	189
HIV/AIDS	179	181	179	172	163	153	142	129	119	111	106	102	100
Malaria	387	381	377	378	359	333	316	244	203	204	191	192	186
Tuberculosis	59	58	57	57	58	59	60	62	62	60	59	59	58
EVD	0	0	0	0	0	0	0	0	0	377	152	0	0

C. Sierra Leone

Category	2005	2006	2007	2008	2009	2010	2011	2012	2013	2014	2015	2016	2017
Total	5,566	5,566	5,526	5,446	5,409	5,332	5,263	5,182	5,034	5,219	5,024	4,846	4,821
VPDs	694	672	589	548	547	534	502	504	462	448	424	386	345
HIV/AIDS	126	146	164	174	183	183	177	170	167	160	151	138	120
Malaria	1,260	1,281	1,298	1,318	1,244	1,143	1,068	962	872	795	811	852	846
Tuberculosis	177	175	173	170	169	169	165	163	158	155	151	146	143
EVD	0	0	0	0	0	0	0	0	0	288	133	0	0

D. Democratic Republic of Congo

Category	2005	2006	2007	2008	2009	2010	2011	2012	2013	2014	2015	2016	2017
Total	48,720	49,163	49,654	50,049	50,473	49,704	49,240	48,743	47,624	46,594	45,460	44,729	44,300
VPDs	6,370	6,214	6,070	6,168	6,016	6,151	6,346	6,219	5,735	5,115	4,679	4,441	4,073
HIV/AIDS	2,660	2,686	2,677	2,641	2,566	2,433	2,296	2,105	1,867	1,642	1,419	1,191	1,000
Malaria	9,408	9,524	9,602	9,238	8,528	7,747	7,025	6,529	6,304	6,283	6,023	6,042	5,998
Tuberculosis	2,301	2,332	2,357	2,397	2,444	2,445	2,443	2,439	2,389	2,363	2,337	2,279	2,227
EVD	0	0	21	2	0	0	0	3	0	5	0	0	1

Supplementary Table S3: DAH/DALYs in EVD affected countries

A. Guinea

Category	2005	2006	2007	2008	2009	2010	2011	2012	2013	2014	2015	2016	2017
Total	5.8	6.4	4.8	6.2	5.8	8.0	6.4	9.8	8.0	35.3	37.2	36.2	22.6
VPDs	3.2	3.9	0.8	5.8	7.7	9.4	3.2	4.6	4.1	6.7	14.0	3.5	1.5
HIV/AIDS	42.8	44.6	29.8	29.8	31.3	31.6	44.7	25.3	64.9	60.7	70.4	155.4	91.5
Malaria	5.0	0.6	4.4	2.2	0.8	14.2	0.3	27.2	10.9	51.4	22.4	70.4	32.7
Tuberculosis	0.1	5.3	6.1	8.1	4.3	10.2	1.9	1.6	8.7	14.6	10.9	15.7	17.1
EVD	—	—	—	—	—	—	—	—	0.0	480.3	1784.4	101943.6	39707334

B. Liberia

Category	2005	2006	2007	2008	2009	2010	2011	2012	2013	2014	2015	2016	2017
Total	10.5	14.5	13.6	35.8	49.4	51.9	55.6	66.3	61.3	146.4	276.8	110.1	75.3
VPDs	6.9	18.2	12.8	20.9	50.5	17.6	25.1	38.5	25.5	7.8	44.9	16.2	23.1
HIV/AIDS	34.5	28.4	52.7	69.2	95.5	126.4	153.8	151.2	192.1	80.4	99.5	173.5	179.5
Malaria	16.2	21.8	3.9	48.0	58.0	87.5	90.1	141.2	110.0	149.4	99.7	125.7	143.3
Tuberculosis	46.9	33.6	2.3	43.9	71.9	62.0	45.2	98.6	57.6	6.1	18.5	22.3	17.9
EVD	—	—	—	—	—	—	—	—	—	560.6	3304.0	490373.9	172528597

C. Sierra Leone

Category	2005	2006	2007	2008	2009	2010	2011	2012	2013	2014	2015	2016	2017
Total	9.2	9.0	9.7	15.0	17.0	21.0	18.7	19.5	23.8	111.6	132.5	71.0	43.1
VPDs	2.0	5.3	11.0	7.2	21.9	19.7	5.6	19.2	14.3	23.2	30.6	10.8	20.2
HIV/AIDS	72.0	47.8	51.4	83.5	88.9	139.3	110.0	126.7	85.4	140.3	51.8	150.1	246.6
Malaria	6.3	6.4	4.4	8.1	5.5	8.1	16.9	4.5	13.9	22.7	8.9	17.4	19.0
Tuberculosis	5.4	6.6	10.9	16.6	4.4	14.6	7.2	4.7	23.8	7.3	11.2	26.0	68.7
EVD	—	—	—	—	—	—	—	—	—	1253.2	3421.3	411813.7	603652128

D. Democratic Republic of Congo

Category	2005	2006	2007	2008	2009	2010	2011	2012	2013	2014	2015	2016	2017
Total	3.6	3.8	3.8	8.8	9.4	10.0	11.5	14.2	14.3	14.5	15.9	16.0	18.5
VPDs	2.0	4.0	3.5	8.4	6.7	10.3	12.7	4.4	13.0	21.2	21.9	16.5	16.9
HIV/AIDS	15.9	15.8	16.2	29.5	34.2	40.6	42.7	77.6	69.6	49.9	61.3	105.4	151.1
Malaria	3.0	1.6	1.5	6.5	13.6	11.5	11.3	30.5	20.7	30.8	28.3	32.3	32.1
Tuberculosis	1.8	4.4	6.3	7.5	5.7	6.6	6.7	15.0	9.1	9.5	18.7	16.7	14.5
EVD	—	—	22.3	65.9	0.0	—	—	0.0	0.0	17.1	1027481.5	—	123.4
