# Supplementary figures and images for: Disparities in Health Financing Allocation among Infectious Diseases in Ebola Virus Disease (EVD)-Affected Countries, 2005–2017
Source: Healthcare (Basel). 2022 Jan 18;10(2):179. doi: 10.3390/healthcare10020179 (PMC8872520; doi:10.3390/healthcare10020179)

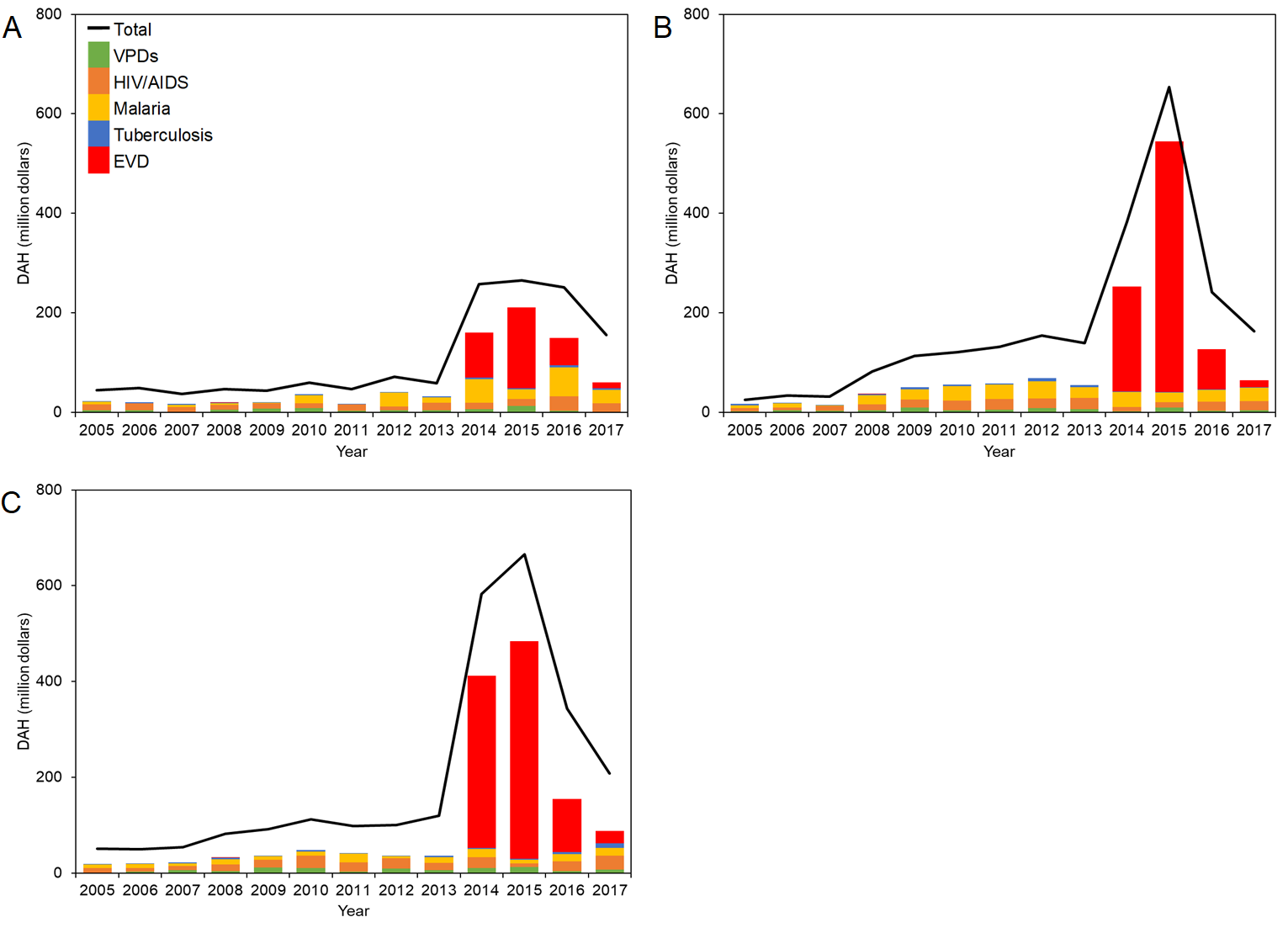

Supplement: Supplementary file 1 [file healthcare-10-00179-s001.zip › shimizu_supfigure S1_9Jan2022.tif]

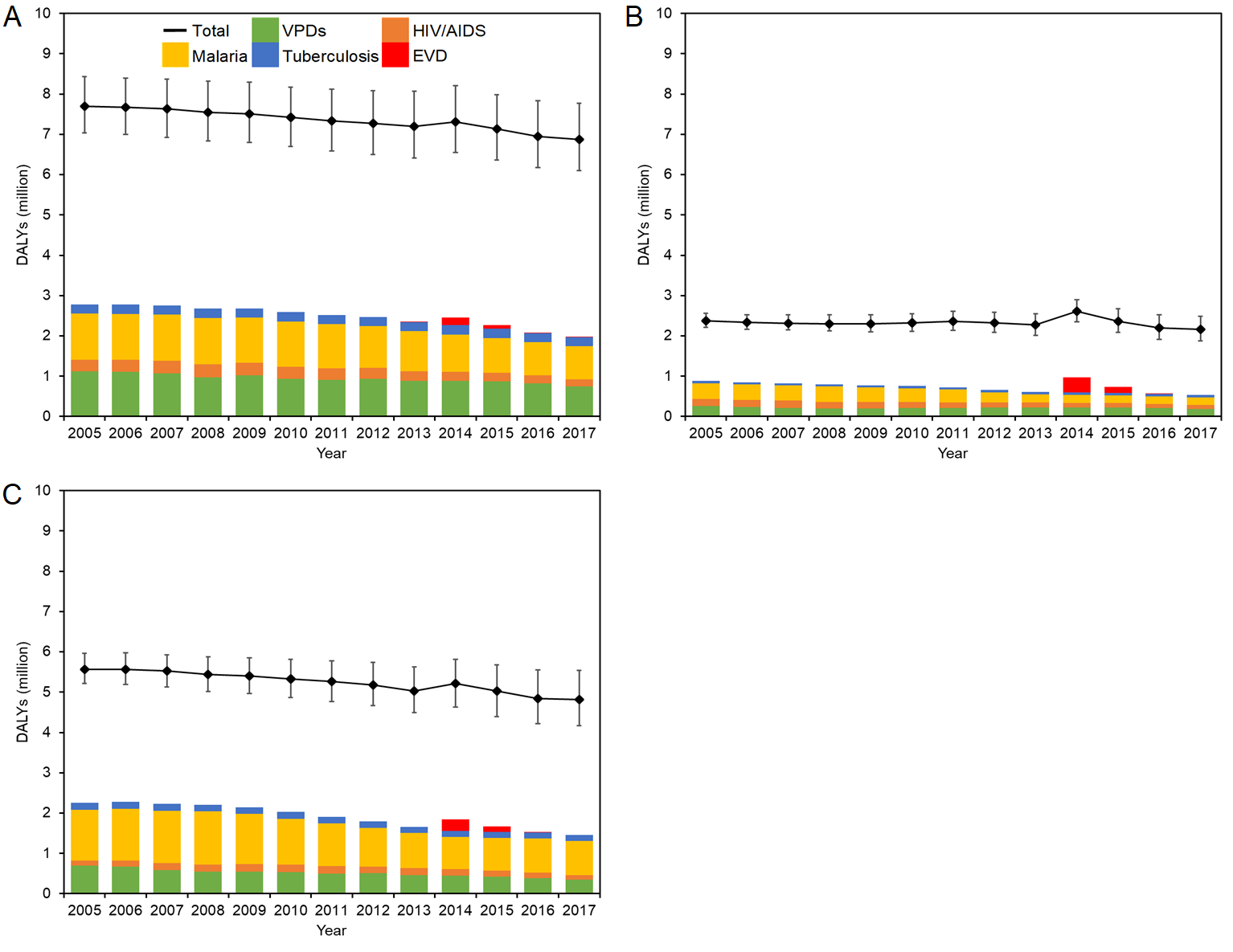

Supplement: Supplementary file 1 [file healthcare-10-00179-s001.zip › shimizu_supfigure S2_9Jan2022.tif]
